# Early extractions of premolars reduce age-related crowding of lower incisors: 50 years of follow-up

**DOI:** 10.1007/s00784-022-04416-x

**Published:** 2022-02-24

**Authors:** Maurits Persson, Nameer Al-Taai, Karin Pihlgren, Anna Westerlund

**Affiliations:** 1 Orthodontics, Department of Odontology, Umeå University, 901 85 Umeå, Sweden; 2 Department of Orthodontics, Public Dental Health Service, 631 88 Eskilstuna, Sweden; 3 Department of Orthodontics, Sahlgrenska Academy, University of Gothenburg, Box 450, 40530 Gothenburg, Sweden

**Keywords:** Class I malocclusion, Incisor irregularity, Serial extraction, Child, Adult, Humans

## Abstract

**Objectives:**

To study the effects of extraction of four premolars, without subsequent orthodontic treatment, on the crowding of lower incisors in subjects between early adolescence and late adulthood, as compared to untreated subjects.

**Materials and methods:**

A total of 45 subjects were included in this study. The extraction group comprised 24 subjects who had all the first premolars removed at a mean age of 11.5 years, to relieve crowding in a class I malocclusion without subsequent orthodontic treatment. The control group had 21 untreated subjects, having a normal occlusion at a mean age of 13.0 years. The participants were documented with dental casts and cephalograms at mean ages of 11.4 and 13.0 years, for the two groups respectively (T1), and at mean ages of 30.9 years (T2) and 61.7 years (T3). Changes in lower incisor crowding were described as changes in “irregularity” and “space deficiency.”

**Results:**

The extraction group showed no changes in the irregularity of the lower incisors and significant improvement of the space deficiency of the lower teeth into late adulthood. While in the control group, both irregularity of the lower incisors and space deficiency of the lower teeth increased significantly into late adulthood.

**Conclusion:**

Lower incisor alignment remains mainly unchanged into late adulthood in subjects who have all their first premolars removed in childhood, as the only treatment to relieve teeth crowding.

**Clinical relevance:**

Severe crowding in a class I occlusion can be solved solely with premolar extraction, allowing for spontaneous adjustments with more stable incisor alignment up to late adulthood.

## Introduction

Crowding is the most frequent malocclusion [1, 2] and is the most common reason why many adults seek orthodontic treatment [2, 3]. Crowding of the front teeth, especially the lower incisors, is considered to be the most pronounced, age-related physiologic change in the dentition [4–8]. These late changes also affect patients who undergo orthodontic treatments [9–12]. Studies have demonstrated that 70–90% of patients experience an unacceptable degree of post-retention crowding [12–15]. Therefore, the preservation of lower incisor alignment is the one of the most challenging tasks in orthodontics. Thus, lifelong retention of alignment of the teeth has been suggested [12, 15, 16], to avoid time-consuming and costly realignments.

However, the mechanisms underlying age-related lower incisor crowding, both in treated and untreated subjects, are still not well understood. Lower incisor crowding has been attributed to multiple factors, including mesial migration of the posterior teeth and lingual inclination of the incisors [9, 17–19].

Extraction of all the first premolars with subsequent orthodontic treatment is the most commonly used method to relieve dental crowding. The significance and timing of extraction as part of the orthodontic treatment for late incisor crowding have been extensively studied. The results reveal no difference in late incisor crowding irrespective of whether the orthodontic treatment is preceded by serial extraction or early or late premolar extraction [12, 14, 20]. In addition, choosing a non-extraction orthodontic treatment has also been shown to result in post-retention crowding [11, 13, 21]. Moreover, a greater increase in age-related incisor crowding has been shown in treated patients compared to untreated subjects in a recent study that analyzed occlusal changes 40 years after premolar extraction and orthodontic treatment [22]. Thus, Sinclair and Little [7, 23] have suggested that orthodontic treatment acts as a promoter of future physiologic changes by shortening the dental arches. Moreover, some studies have recommended that the lower incisors should be retained in their original position, so as to minimize the risk of relapse [24, 25]. Consequently, it is interesting to investigate whether patients who initially had incisor crowding and were treated with extraction without subsequent orthodontic treatment also developed more-pronounced long-term incisor crowding, as compared to untreated subjects who had normal occlusion. The significance of early premolar extraction, without subsequent orthodontic treatment for late incisor crowding, has been scarcely studied [26–28]. The concept has been advocated to reduce or eliminate the need for treatment with a fixed appliance [29]. The space acquired through premolar extractions, in accordance with the principles of serial extraction methods, may facilitate spontaneous alignment of lower arch incisor crowding and stability up to early adulthood [27]. In that study, the spontaneous closure of extraction gaps resulted in an improvement in the malocclusion index similar to that seen in non-treated, normal occlusion cases [27].

Furthermore, the significance of unclosed premolar extraction gaps for late incisor crowding has not been explored to date. The aim of the present study was, therefore, to investigate the physiologic changes in the mandibular incisors’ area that occurred from early adolescence to late adulthood in patients with a class I crowding malocclusion who were treated in the mixed dentition by extraction of all the first premolars without subsequent orthodontic treatment. We compared the outcomes for these patients with those of an untreated group with an initial normal occlusion.

## Materials and methods

### Study design and subjects

The study is a 50-year, longitudinal, case–control study. The subjects, established as two groups, in the 1960s, were all patients in the Public Dental Health Care system in Umeå, Sweden. The exclusion criteria for the present study were as follows: missing teeth or prosthodontic treatment, including the teeth mesial to the lower second molars; and using mandibular advancement devices for sleep apnea.

The present follow-up study was approved by the Regional Ethical Board in Umeå University, Sweden (registration no. 2012–410-31 M). Written informed consent was obtained from all participants.

#### Extraction group

**Fig. 1:**
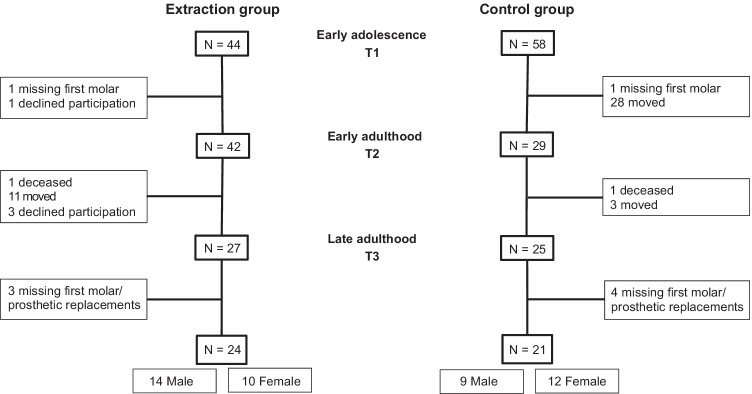
Flowchart of the subjects who participated in the study, indicating dropouts. Overall, 24 and 21 subjects were included in the extraction group and control group, respectively, at T1, T2, and T3

The extraction group consisted of 24 subjects who had all their first premolars removed at a mean age of about 11.5 years (T1) to treat a class I space deficiency malocclusion. No orthodontic treatment was undertaken due to inadequate specialist resources, thereby allowing potential spontaneous alignment of arches and closure of extraction gaps over time [27]. The mean ages for the 24 included subjects in the extraction group were 11.4, 30.4, and 61.8 years at T1, T2, and T3, respectively (Fig. 1).

#### Control group

The control group consisted of 21 subjects who were classified as having a normal occlusion at the age of 13 years (T1). The mean ages for the 21 included subjects in the control group were 13, 31.5, and 61.7 years at T1, T2, and T3, respectively (Fig. 1).

### Data collection

Clinical examinations and dental cast documentations of the subjects in both groups were recorded at the three timepoints of T1, T2, and T3. In addition, digital cephalometric analyses were performed at T1, T2, and T3 for the extraction and control groups. All examinations were made at the Department of Orthodontics at the School of Dentistry in Umeå. A digital sliding caliper (Velleman, 0.01 mm) was used for linear measurements on casts. All the measurements were performed by one orthodontist. The cephalometric analysis was performed according to a previous study [30].

### Study variables

#### Lower incisor crowding

**Fig. 2:**
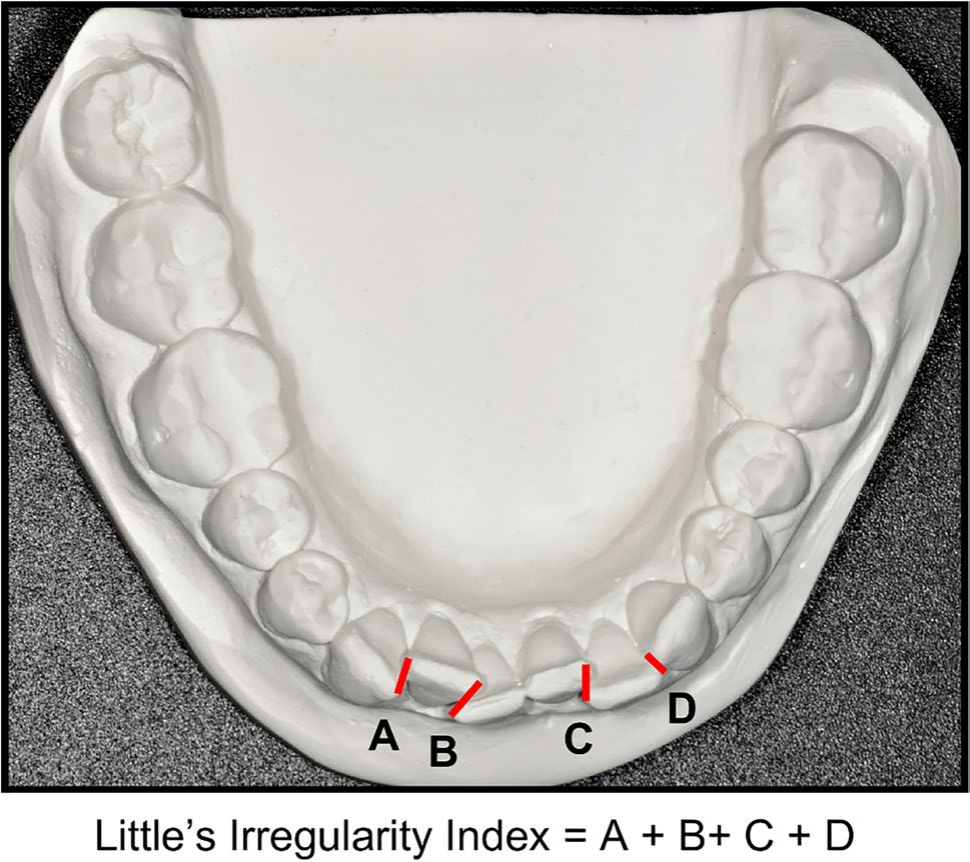
Irregularity Index (mm) was defined by Little as the summed displacement of adjacent anatomic contact points of the six mandibular anterior teeth

- *Irregularity*, recorded according to Little’s Irregularity Index [31] (Fig. 2).
- *Space deficiency* (tooth size-arch length discrepancy; TSALD), as described by Bishara [32]. TSALD was measured for the six anterior teeth (TSALDant) and for the whole arch mesial to the first molars (TSALDtot) [9]. A positive value for TSALD indicates spacing, while a negative value indicates a space deficiency. When needed, the tooth width of an unerupted tooth at T1 was measured on the casts of a succeeding documentation.

#### Dentoalveolar changes

**Fig. 3:**
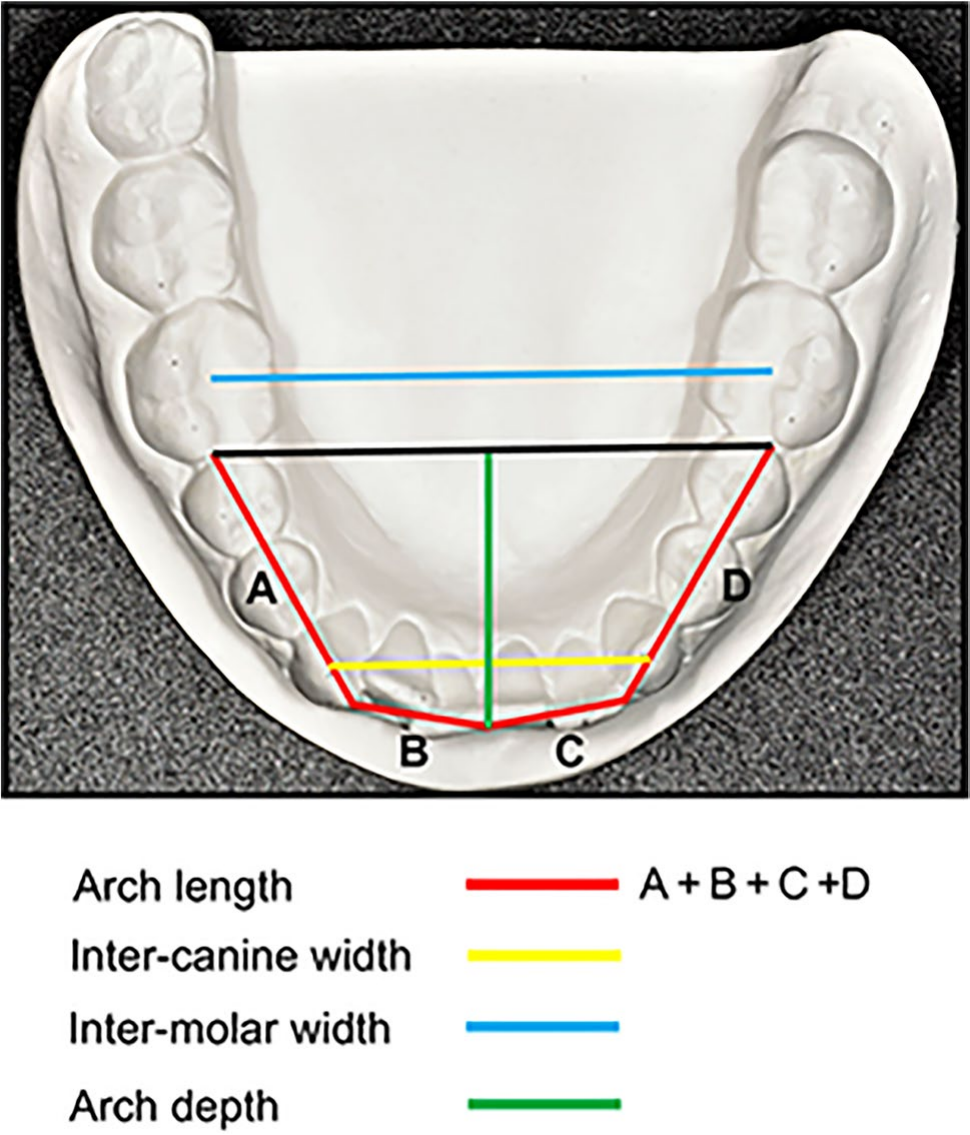
Arch length (mm) was defined as the total length of the posterior and anterior segments mesial to the first permanent molars. The distance between the tips of the lower canines represents the inter-canine width (mm). The distance between the central fossa of the lower first molars represents the inter-molar width (mm). Arch depth (mm) was acquired by measuring the perpendicular distance, at the midline, from the labial surface of the lower central incisors to the mesial surface of the lower first molars

- Sagittal occlusal relation
- Overjet
- Overbite
- Sum of lower incisor widths
- Total lower arch length between first molars (*arch length*) (Fig. 3)
- Lower arch depth to first molar line (*arch depth*), (Fig. 3)
- Lower inter-molar arch width at first molars (*inter-molar width*) (Fig. 3)
- Lower inter-canine arch width (*inter-canine width*) (Fig. 3)

#### Cephalometric changes

- Lower incisor inclination in relation to the mandibular plane (L inc/ML)
- Linear distance between the lower incisor and the A-Pogonion plane (L inc-A-Pog)
- Anterior facial height; linear distance between the Nasion and Menton landmarks (N-Me)
- Posterior facial height; linear distance between the Sella and Gonion landmarks (S-Go)

### Data analysis

#### Method error

The intra-observer error of measurements made on casts was assessed using duplicate measurements, performed 2 months apart on extraction casts at T1. The error of the method was calculated using Dahlberg’s formula, with a mean value of 0.43 mm for all linear measurements. The mean error of the mandibular Irregularity Index measurements was 0.52 mm, and that of the TSALDtot measurements was 0.43 mm (Table 1).

**Table 1:** The error of the method as calculated using Dahlberg’s formula

Overjet	0.47
Overbite	0.45
Total lower incisor widths	0.55
IrregI	0.52
Arch length right anterior	0.36
Arch length left anterior	0.23
Arch length right posterior	0.33
Arch length left posterior	0.57
Inter-canine width	0.46
Inter-molar width	0.39
Arch depth	0.46
TSALDtot	0.43
**Mean**	**0.43**
**Min**	**0.23**
**Max**	**0.57**

Bold entries highlight the statistically significant value

The method error for cephalometric measurements was previously described by Al-Taai et al. [30].

#### Statistical analysis

Statistical tests of crowding and dentoalveolar changes with age within the groups were tested using the non-parametric Wilcoxon signed-rank test, as several of the variable recordings followed a skewed distribution. Tests of differences between groups were performed using the Mann–Whitney *U*-test. Associations between incisor crowding (Irregularity Index and TSALDant) and arch length, width, and depth were assessed using mixed effects models with “developmental period” as a fixed factor and “subjects” as random effects.

All statistical analyses were performed using the R ver. 4.0.0 software (R Core Team 2020) and the significance level was set at 0.05.

## Results

### General observations

Out of 45 cases in the two groups, 44 cases remained in the original class I molar relation, showing less than half cusp width changes from T1 to T3. One patient in the extraction group developed a class III molar relation but retained the normal incisor relation.

The values for the linear dentoalveolar changes were low in both groups during the adult period (Table 2).

**Table 2:** Dentoalveolar changes (mm) at the three developmental periods for the subjects in the two groups. Shown are the median values (1st and 3rd quartiles), including the *p*-values from the Wilcoxon signed-rank test of changes between T2 and T3 within the groups, and the *p*-values from the Mann–Whitney *U*-test of sample group differences in the changes over time

Period/variable	Extraction group				Control group				*p*-value for group comparison		
	**T1**	**T2**	T3	*p* T2 vs T3	T1	T2	T3	*p* T2 vs T3	T1–T2	T1–T3	T2–T3
Overjet	3.9 [2.8, 5.0]	3.3 [2.4, 4.1]	3.4 [2.4, 4.3]	0.897	3.0 [2.5, 3.4]	2.5 [2.4, 2.9]	2.6 [2.0, 3.2]	0.808	0.652	0.817	0.682
Overbite	3.8 [3.0, 4.4]	2.8 [2.2, 3.2]	2.7 [1.8, 3.2]	0.269	3.0 [2.4, 3.7]	2.8 [2.4, 3.2]	2.4 [1.3, 3.0]	0.062	0.188	0.131	0.363
Total lower incisor widths	24.8 [23.7, 25.6]	24.4 [23.5, 25.7]	24.4 [23.7, 24.9]	0.203	23.3 [22.7, 23.9]	22.8 [21.5, 24.0]	22.5 [21.6, 22.9]	0.289	0.503	0.450	0.674
Arch length	61.5 [59.1, 62.9]	52.5 [49.8, 54.6]	51.2 [49.2, 53.3]	0.178	64.2 [62.7, 65.6]	62.3 [60.5, 63.3]	61.1 [58.8, 63.0]	**0.001**	** < 0.001**	** < 0.001**	0.180
Arch depth	33.1 [32.3, 33.9]	27.3 [25.9, 28.2]	27.0 [26.1, 27.9]	0.327	32.9 [32.2, 33.6]	31.1 [30.1, 32.6]	30.3 [28.9, 31.8]	** < 0.001**	** < 0.001**	** < 0.001**	**0.026**
Inter-molar width	39.8 [38.1, 41.3]	38.3 [36.7, 39.8]	38.1 [36.2, 41.0]	0.639	42.0 [40.9, 43.5]	42.9 [40.5, 45.2]	42.9 [41.3, 43.8]	0.779	** < 0.001**	** < 0.001**	0.948
Inter-canine width	26.7* [25.3, 27.7]	25.8 [25.1, 26.8]	25.3 [24.0, 26.6]	**0.019**	25.8 [24.9, 26.7]	25.0 [24.3, 26.0]	24.5 [23.5, 25.5]	**0.006**	0.584	0.831	0.633

Bold entries highlight the statistically significant value

^*^N = 17 due to non-erupted or canine under eruption

The incisor relations expressed by the overjet and overbite recordings were stable with no significant differences within the groups or between the groups over time (Table 2).

The total reduction in incisor tooth width with age from T1 to T3 was − 0.4 mm in the extraction group and − 0.8 mm in the control group, with no significant differences within or between the groups over time (Table 2).

### Lower incisor crowding

**Fig. 4:**
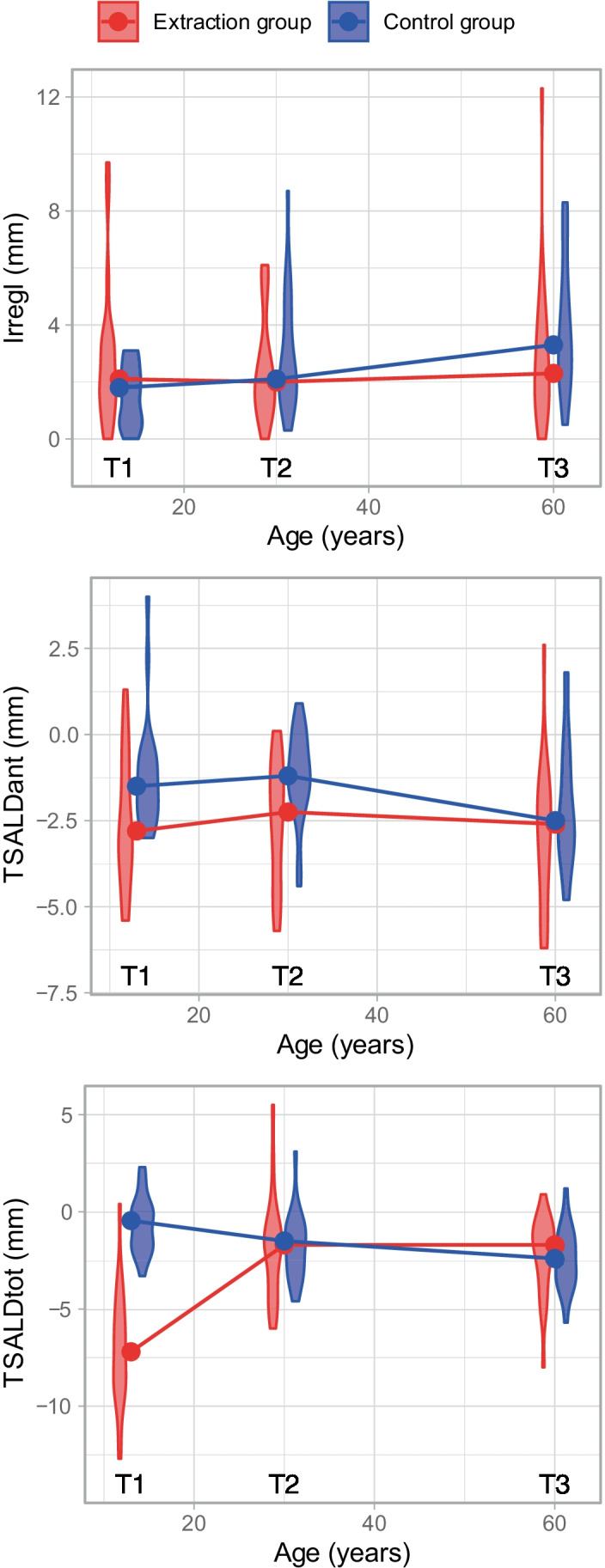
Graphic illustration of the median levels (circles) of the Irregularity Index (IrregI), anterior (TSALDant), and total (TSALDtot) tooth size/arch length discrepancy at developmental periods T1 to T3 for the premolar extraction group and the control group. Violin plots reveal the distribution form and dispersion of data within the groups at the different periods

The changes in the Irregularity Index of the extraction group were non-significant from early adolescence to early adulthood (T1–T2), as well as early to late adulthood (T2–T3). In contrast, the Irregularity Index increased significantly during the same periods in the control group. Significant differences in the Irregularity Index between the groups were found for the periods from early adolescence to early adulthood (T1–T2) and early adolescence to late adulthood (T1–T3), with the extraction group showing a lower Irregularity Index score than the control group. Despite this, no significant differences were noted between the groups for the late period from early to late adulthood (T2–T3) (Tables 3 and 4, Fig. 4).

**Table 3:** Median (1st and 3rd quartiles) of the Irregularity Index (IrregI), anterior (TSALDant), and total (TSALDtot) tooth size/arch length discrepancy at the three developmental periods for the two groups

Period/variable	Extraction group			Control group		
	T1	T2	T3	T1	T2	T3
IrregI (mm)	2.1 [1.4, 3.2]	2.0 [1.2, 3.1]	2.3 [1.2, 3.9]	1.8 [0.5, 2.3]	2.1 [1.7, 4.3]	3.3 [2.3, 5.3]
TSALDant (mm)	− 2.8 [− 3.6, 3.2]	− 2.2 [− 4.3, 3.1]	− 2.6 [− 4.2, 3.9]	− 1.5 [− 2.2, 2.3]	− 1.2 [− 1.7, 4.3]	− 2.5 [− 3.2, 5.3]
TSALDtot (mm)	− 7.2 [− 8.8, − 5.5]	− 1.7 [− 3.7, − 1.0]	− 1.7 [− 3.3, − 0.8]	− 0.4 [− 1.8, 0.0]	− 1.5 [− 3.1, − 0.8]	− 2.4 [− 3.4, − 1.4]

**Table 4:** Median (1st and 3rd quartiles) of the changes (mm) in the Irregularity Index (IrregI), anterior (TSALDant), and total (TSALDtot) tooth size/arch length discrepancy, including the *p*-values from the Wilcoxon signed-rank test of the changes within groups, and the *p*-values from the Mann–Whitney *U*-test of sample group differences in the changes

Variable	Age period	Extraction group			Control group			*p*-value for group comparison
		Median	IQR	*p*-value	Median	IQR	*p*-value	
IrregI	T2–T1	− 0.40	[− 1.1, 1.0]	0.709	1.15	[0.5, 2.9]	**0.001**	**0.009**
IrregI	T3–T1	0.30	[− 1.2, 1.7]	0.728	2.40	[0.8, 3.6]	** < 0.001**	**0.004**
IrregI	T3–T2	0.00	[− 0.8, 1.3]	0.603	0.60	[− 0.1, 1.8]	**0.029**	0.255
TSALDant	T2–T1	− 0.40	[− 2.0, 1.8]	0.808	0.00	[− 1.2, 1.5]	0.852	0.922
TSALDant	T3–T1	− 0.10	[− 1.9, 1.1]	0.475	− 1.25	[− 2.2, 0.3]	0.089	0.381
TSALDant	T3–T2	− 0.55	[− 1.7, 0.2]	0.123	− 1.00	[− 1.9, 0.3]	0.060	0.517
TSALDtot	T2–T1	5.10	[3.0, 7.0]	** < 0.001**	− 0.80	[− 2.0, − 0.4]	** < 0.001**	** < 0.001**
TSALDtot	T3–T1	4.70	[2.8, 7.2]	** < 0.001**	− 1.50	[− 2.2, − 1.0]	** < 0.001**	** < 0.001**
TSALDtot	T3–T2	0.30	[− 1.1, 0.8]	0.808	− 0.50	[− 1.4, 0.4]	**0.027**	0.125

Bold entries highlight the statistically significant value

Although TSALDant increased more in the control group, particularly during early to late adulthood (T2–T3), no statistically significance difference for either group was found over time, nor were there any differences between the groups (Tables 3 and 4).

The TSALDtot values showed a significant decrease in extraction group from early adolescence to early adulthood (T1–T2), as well as to late adulthood (T1–T3). In the control group, the TSALDtot values increased significantly from early adolescence to early adulthood (T1–T2) and to late adulthood (T2–T3). There were significant differences between the groups in terms of the TSALDtot values when comparing changes during the age periods of T1–T2 and T1–T3, but not when comparing changes as adults separately (T2–T3) (Tables 3 and 4, Fig. 4).

### Dentoalveolar changes

No significant changes in arch length or in arch depth were found from early to late adulthood in the extraction group (T2–T3). This contrasts with the control group, where arch length and arch depth decreased significantly during this period. Arch length and arch depth, as well as inter-molar width, differed significantly between the two groups from early adolescence to early adulthood (T1–T2) and early adolescence to late adulthood (T1–T3). Only changes in arch depth demonstrated a significant difference between the groups during the adult period (T2–T3) (Table 2). In both groups, the inter-canine width decreased significantly from early to late adulthood (T2–T3). However, there were no significant differences in inter-canine width changes between the groups over time (Table 2).

Testing for a correlation between the variable changes and groups during the adult period (T2–T3), and using the Irregularity Index as the independent variable, showed that only arch depth was significantly correlated and limited to the extraction group (Table 5).

**Table 5:** Spearman correlations to changes in the Irregularity Index (IrregI) between T2 and T3

Extraction group	rho_extraction	p_extraction
TSALDtot*	− 0.1337108	0.533
TSALDant	− 0.0139312	0.948
Arch length	− 0.0757239	0.731
Arch depth	− 0.4639825	0.030
Inter-canine width	− 0.1841934	0.389
Control group	rho_control	p_control
TSALDtot*	− 0.4230020	0.056
TSALDant	0.0579239	0.803
Arch length	− 0.3372358	0.135
Arch depth	− 0.2864109	0.208
Inter-canine width	− 0.3762602	0.093

^*^Total tooth size/arch length discrepancy for the whole arch mesial to the first molars

With TSALDant chosen as an independent variable, a non-significant correlation was shown in both groups (Table 6).

**Table 6:** Spearman correlations to changes in the tooth size/arch length discrepancy for the six anterior teeth (TSALDant) between T2 and T3

Extraction group	rho_extraction	p_extraction
TSALDtot*	0.0104439	0.961
IrregI	− 0.0139312	0.948
Arch length	0.1552534	0.479
Arch depth	0.1181308	0.601
Inter-canine width	0.3297803	0.116
Control group	rho_control	p_control
TSALDtot*	0.2816264	0.216
IrregI	0.0579239	0.803
Arch length	0.3144532	0.165
Arch depth	0.0133420	0.954
Inter-canine width	− 0.1100261	0.635

^*^Total tooth size/arch length discrepancy for the whole arch mesial to the first molars

### Cephalometric changes

No significant differences in incisor inclination and position (L inc/ML and L inc-A-Pog) were found between the groups throughout the observation period. In both groups, the anterior and posterior facial heights (N-Me and S-Go) increased from early adolescence to early adulthood (T1–T2) and from early adolescence to late adulthood (T1–T3). These increases differed significantly between the groups in the corresponding periods. However, there were no significant differences between the groups with respect to the anterior and posterior facial heights from early to late adulthood (T2–T3) (Table 7).

**Table 7:** Median (1st and 3rd quartiles) of the changes (mm) in the distance between the lower incisor and the A-Pogonion line (L inc-A-Pog), anterior facial height (N-Me) and posterior facial height (S-Go), and changes (°) in the inclination of the lower incisor with respect to the mandibular plane (L inc/ML), including the *p*-values obtained from the Wilcoxon signed-rank test of the changes within groups, and the *p*-values from the Mann–Whitney *U*-test of sample group differences in the changes

Variable	Age period	Extraction group		Control group		*p*-value for group comparison
		Median	IQR	Median	IQR	
L inc-A-Pog	T2–T1	− 0.70	[− 1.6 to 0]	− 0.2	[− 1.1 to 0.2]	0.318
L inc-A-Pog	T3–T1	− 0.80	[− 1.8 to 0.2]	− 0.3	[− 1.3 to 0.1]	0.513
L inc-A-Pog	T3–T2	− 0.10	[− 0.7 to 0.1]	− 0.1	[− 0.6 to 0.4]	0.761
L inc/ML	T2–T1	0.10	[− 2.2 to 4.8]	3.1	[− 0.1 to 5.1]	0.329
L inc/ML	T3–T1	0.10	[− 4.3 to 3.2]	0.5	[− 2.3 to 2.4]	0.651
L inc/ML	T3–T2	− 1.65	[− 3.1 to 0.2]	− 1.3	[− 3.3–0.3]	0.715
N-Me	T2–T1	12.90	[9.6 to 16.6]	10.3	[8.2–12.7]	**0.047**
N-Me	T3–T1	14.20	[11–16.8]	9.3	[8.7–13.1]	0.050
N-Me	T3–T2	0.45	[− 1.7 to 1.3]	1.1	[− 0.8 to 1.4]	0.560
S-Go	T2–T1	13.50	[6.5–17.5]	8.3	[6–10.8]	**0.041**
S-Go	T3–T1	13.20	[7.1–16.5]	5.9	[4.2–10.4]	**0.003**
S-Go	T3–T2	− 1.05	[− 2–0.1]	− 1.8	[− 2.4 to 1]	0.101

Bold entries highlight the statistically significant value

## Discussion

Our study shows that lower incisor alignment remains essentially unchanged from early adolescence to late adulthood in patients who have their first premolar extracted as the sole treatment for crowding in Angle class I malocclusion. This contrasts with the development of severe incisor crowding in the cohort of normal occlusion cases during the corresponding developmental period.

The patient sample was initially documented in the 1960s as part of a longitudinal evaluation of limited treatment for dental crowding in children [27]. While the concept of premolar extraction was intended to be in line with classical serial extraction [29], in several of our extraction cases, the methodologically planned removal of primary teeth was disrupted due to late referrals and therefore lacked potential extraction-related alignment of the anterior teeth. No long-term studies of premolar extractions that allow spontaneous alignment up to late adulthood have been conducted to date. In follow-up studies of serial extraction treatment, the patients were followed for 2, 3, and 7 years, respectively, and up to 20 years of age [26, 28, 33]. Therefore, direct comparisons with earlier serial extraction studies must be made with caution.

Development of lower incisor crowding with age has been described mainly using Little’s Irregularity Index [31]. However, as crowding is associated with changes in arch length, the tooth size/arch length discrepancy must also be considered [9, 12, 32]. Moreover, since these study populations are uncommon, it is of value to use different variables to allow for comparisons between studies.

As for the Irregularity Index, no significant changes with age were found for the TSALDant variable in the extraction group, demonstrating stability of the available arch space in the anterior segment. Moreover, a significant improvement in the TSALDtot variable was observed over time, particularly from early adolescence to early adulthood, due to the premolar extraction. An initial decrease in incisor crowding, as is usually expected in serial extraction, may be obscured in our data because these changes with age are unlikely to be linear. The large individual variation in lower crowding, as evidenced by TSALDtot values close to zero, is explained by cases with severe crowding in the upper arch, providing arguments for extraction treatment.

The significant aggravation of crowding with age observed in the control group, as described by the Irregularity Index as well as the TSALDtot variable, is in accordance with the results of several studies of untreated groups [5, 6, 8, 23, 32]. However, the significant increases in the Irregularity Index and TSALDtot from early adulthood to late adulthood in the control group contrast with an earlier study showing that such a deterioration is less significant after 30 years of age [11].

Our results are not in agreement with the observation made by Woodside and coworkers, who found no significant difference in incisor crowding between serial extraction cases and a control group at 7 years of follow-up [33]. It is noteworthy that patients who were treated with serial extraction in that study were also given “minor orthodontic treatment.” Long-term evaluation of early extraction of premolars and subsequent orthodontic treatment have been linked to a significant increase in the degree of crowding with age, even though the starting point in these cases was aligned incisors [12, 14].

Arch length and arch depth were stable from early to late adulthood in the extraction group. In contrast, the control group showed a significant decrease in arch length and arch depth from early to late adulthood. Results similar to those seen in our untreated control group have been described in several earlier longitudinal studies of an untreated sample, with changes in size and shape of the dental arches for time spans that included late adulthood [5, 6, 8, 23, 32]. We observed no significant long-term effect on incisor inclination following extraction compared to the control group. Consequently, the long-term increase in lower incisor crowding in the control group probably reflects mesial migration of the posterior teeth rather than incisor inclination. In a previous study, however, lower incisor irregularity was observed to increase as the distance between the lower incisor and the A-Pog plane increased [34]. This can be attributed to the fact that the majority of the patients had a class II malocclusion [34].

The observed stability of the incisor relations, as expressed by overjet and overbite, is also in accordance with earlier studies of untreated cases up to late adulthood [8, 32, 35]. The slight decrease in inter-canine width seen in both groups, and with significance for the whole observation period in the control group, appears to be in accordance with previous observations of changes occurring with age in untreated subjects [8, 32].

Correlations between increases in lower incisor irregularity and decreases in lower arch length and inter-canine width have previously been shown for untreated subjects [8, 32, 35]. However, no such correlations for any of studied variables have been shown in the control group in the present study.

The stable arch depth was the only variable in the extraction group that correlated with unchanged incisor irregularity during the tested adult period. We propose that the maintained arch depth is the result of residual premolar extraction spaces, which may accelerate the early mesial migration of posterior teeth and/or dampen the effect of late changes in incisor alignment.

In addition, lower incisor crowding has been associated with facial divergence [9, 18, 34]. However, that association was not observed in the present study, as both groups showed increases in the anterior and posterior facial heights from adolescence to early adulthood, and slight decreases in the corresponding facial heights from early to late adulthood.

A limitation linked to the interpretation of our longitudinal data is that the described dimensional changes are unlikely to be linear and, similar to changes in irregularity discussed above, may vary during the follow-up period. The long follow-up period for these subjects is an indication that there are small changes occurring from 30 to 62 years of age in patients with class I malocclusion. Future research may add to this knowledge. Moreover, it would be interesting to have additional two groups to compare with: (1) a group with crowding treated by premolar extraction and orthodontic appliance with non-actively closed extraction gaps and (2) a group with crowding treated by premolar extraction and orthodontic appliance with actively closed extraction gaps. However, even without these groups, the two presented cohorts in this study are unique.

It has been hypothesized that orthodontic mechanotherapy acts as an accelerator of future physiologic changes [23], thereby placing the teeth in an unstable position that results in post-retention relapse [12, 14], which affects incisor irregularity [33], and that lower arch crowding is related to mesial migration of the first molar [36]. Based on the results of this study, we propose that for patients with normal occlusion and a space deficiency of ≥ 7 mm, the first premolars can be extracted when they erupt, without further orthodontic interventions. However, if the upper and/or the lower incisors require alignment with orthodontic appliances, it may be advantageous to leave the residual extraction spaces open, so as to counteract mesial drift of the molars and subsequent incisor crowding in late adulthood. Whether or not early extraction of premolars will prevent aggravation of lower incisor crowding, also in the normal bite without early crowding, is not known. It is neither ethically nor therapeutically indicated to extract premolars and create gaps, in an otherwise perfect occlusion, in an attempt to achieve a less-irregular lower front later in life.

## Conclusion

- Lower incisor alignment remains mostly unchanged into late adulthood in a cohort of patients with crowding in Angle class I malocclusions treated solely with first premolar extraction, in contrast to the significant increase in lower incisor irregularity in an untreated cohort that was initially classified as having normal occlusion.
- Although minor changes in arch morphology indicate a more-compressed and a shorter dental arch in both groups during adulthood, significant reductions in arch length and arch depth variables are found only in the control group. The non-significant changes in the Irregularity Index in the extraction group correlate with unchanged arch depth.
- We show that severe crowding in a class I occlusion can be solved solely with premolar extraction, allowing for spontaneous adjustments with more stable incisor alignment up to late adulthood.
